# Protracted abstinence in males with an opioid use disorder: partial recovery of nucleus accumbens function

**DOI:** 10.1038/s41398-022-01813-4

**Published:** 2022-02-26

**Authors:** Serenella Tolomeo, Alex Baldacchino, Nora D. Volkow, J. Douglas Steele

**Affiliations:** 1 Social and Cognitive Computing Department, Institute of High Performance Computing, Agency for Science, Technology and Research, Singapore, Singapore; 2 Division of Population and Behavioral Science, Medical School, University of St Andrews, St Andrews, UK; 3 National Institute on Drug Abuse, Bethesda, MD 20892 USA; 4 Division of Imaging Science and Technology, Medical School, University of Dundee, Dundee, UK

**Keywords:** Addiction, Human behaviour

## Abstract

Opioid use disorder (OUD) affects more than 27 million people globally accounting for more than 300,000 deaths annually. Protracted abstinence among individuals with OUD is rare due to a high relapse rate among those not receiving medications for OUD. Extensive preclinical studies form the basis of the allostasis theory, which proposes long-lasting functional brain abnormalities that persist after opioid withdrawal and contribute to relapse. Few studies have tested the allostasis theory in humans using neuroimaging. Here, we used fMRI and an instrumental learning task to test allostasis theory predictions (ATP) of functional abnormalities in both positive valence (PVS) and negative valence (NVS) accumbens systems in OUD patients with protracted abstinence (*n* = 15), comparing them with OUD patients receiving methadone treatment (MT) (*n* = 33), and with healthy controls (*n* = 23). As hypothesized, protracted abstinence OUD patients showed incomplete recovery of nucleus accumbens function, as evidenced by the blunted response to aversive events (NVS) during negative reinforcement, as observed in MT patients. In contrast, their accumbens response to rewarding events (PVS) during positive reinforcement was similar to that of controls and different from that in MT patients whose response was blunted. Protracted abstinence OUD patients also showed improvements in depression symptoms compared to MT patients. Residual depressive symptoms and pre-MT intravenous drug measures were associated with worse accumbens function in protracted abstinence. These results support the ATP of long-lasting dysfunction of NVS after withdrawal and show preliminary evidence of recovery of PVS function with protracted withdrawal. Therapeutic strategies that target NVS may facilitate recovery.

## Introduction

Opioid use disorder (OUD) affects ~27 million people globally with more than 300,000 deaths annually [1]. The COVID-19 pandemic, alongside a rise in misuse of synthetic opioids, has worsened the situation, with the US Centre for Disease Control estimating an increase of ~50% in opioid overdoses deaths during the pandemic in the United Sates [2]. A better understanding of the mechanisms, which underlie the development, maintenance, and recovery from OUD, could help improve the treatments of OUD worldwide [1].

The Research Domain Criteria (RDoC), which were developed by the National Institute of Mental Health (NIMH), link subjective symptoms to the relevant brain systems [3]. Central to OUD is the positive valence system (PVS), which processes rewarding information, and the Negative Valence System (NVS), which processes information about aversive events (e.g., loss and pain) [4]. We recently reported a study [5] on binge alcohol drinking that used RDoC to test the allostasis theory [6], which proposes that increased chronic negative reinforcement drives drug-taking and relapse [7]. Here, we applied the same approach to investigate OUD patients who were in protracted abstinence. Notably, the investigation of OUD patients in protracted abstinence is crucial because of their high relapse rates [1] and high overdoses risks [8]. However, very few studies have examined brain function in OUD patients during protracted abstinence as such patients, because of their rarity, are challenging to recruit. Identifying characteristics associated with treatment success among abstinent previously opioid-dependent individuals is currently a priority to help guide personalized interventions for OUD.

**Fig. 1:**
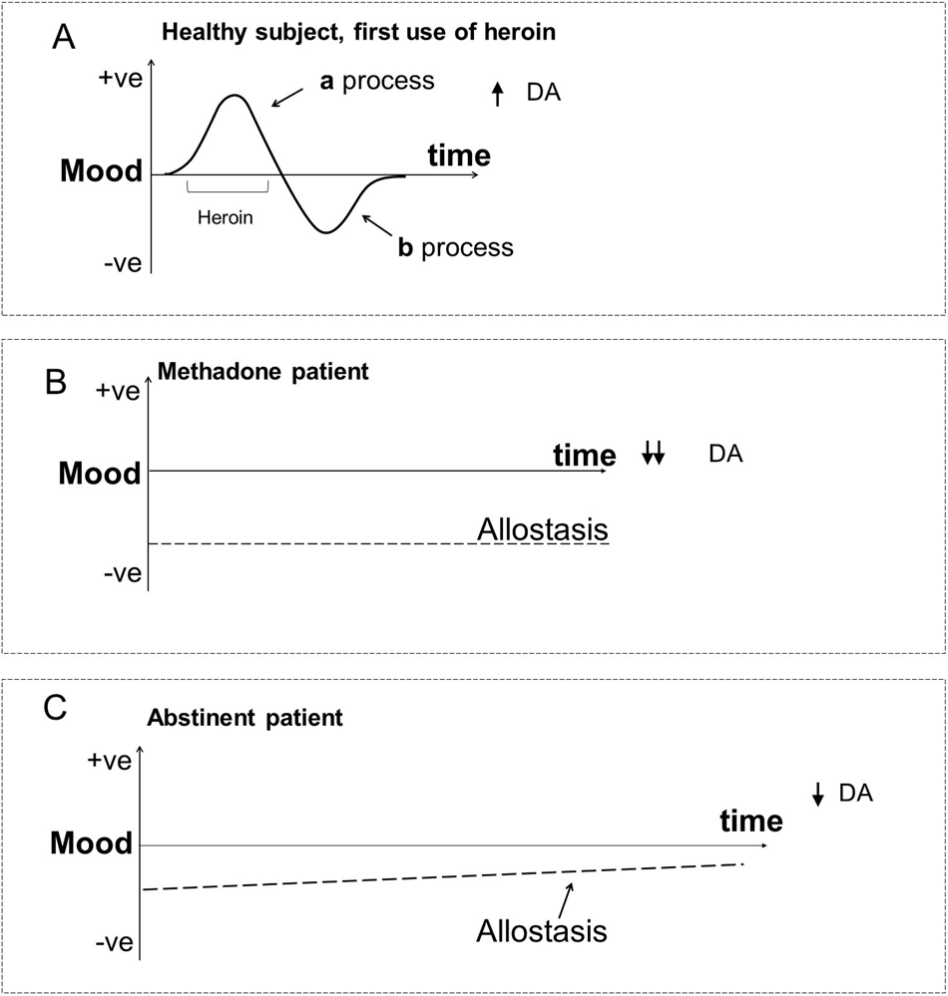
Allostasis theory predictions for opioid dependence and abstinence. **A** First episode of heroin use with positive (+) mood (“a process”=PVS) followed by post-intoxication negative (−) mood (“b process”=NVS). **B** With repeated episodic use of heroin followed by methadone treatment, positive mood change diminishes, and the depth of negative mood increases. The allostatic downward shift in baseline mood is shown by the dashed line. **C** With abstinence following dependence there is slow post-withdrawal partial recovery from the allostatic change that occurred during opioid dependence. The allostatic change is shown by the dashed line [7]. DA dopamine.

The allostasis theory [6] proposes a balance of opponent processes in response to natural (i.e., non-drug stimuli such as social events) and drug-induced changes in effect [7]. As illustrated in Fig. 1A, an initial stimulus (e.g., first exposure to heroin) transiently increases positive mood (“a process”), then is followed by a period of negative mood (“b process”). With repeated drug exposure, the positive “a process” diminishes and the “b process” is enhanced. Additionally, as shown in Fig. 1B, if there is insufficient time for mood to return to homeostatic baseline between episodes of drug use, baseline mood shifts downwards; “allostasis” [6]. In drug dependence (Fig. 1B), reward function (PVS) is blunted and the stress response (NVS) sensitized, impacting responses to both drug and non-drug (e.g., social) stimuli. In opioid dependence, the enhanced “b process” mostly reflects acute drug withdrawal, which depending on the severity lasts 3–10 days in humans [9]. In preclinical models of human addiction, protracted abstinence has also been linked to increased brain reward thresholds and increases in anxiety-like behavior persisting long after acute withdrawal [10], with negative effect particularly important in protracted abstinence [11]. Lower pain tolerance exacerbated by negative emotional states has been reported in OUD individuals during acute (24–72 h) and protracted abstinence (average 30 months) [12]. Repeated and intermittent activation of brain reward circuits by opioids engages ‘anti-reward’ circuits that drive aversive negative emotional states [1]. In turn, the enhancement of negative reinforcement (e.g., avoidance of anxiety or pain) contributes to relapse and the persistence of OUD [13].

Previously we reported PVS blunting and abnormal NVS responses in methadone-treated (MT) patients with a heroin use disorder [14]. It is unclear whether prolonged abstinence in humans results in full or partial recovery from OUD-associated brain changes. Building on pre-clinical studies of protracted abstinence in animals [15], here we used functional magnetic resonance imaging (fMRI) to test the allostasis theory hypotheses that protracted abstinence in OUD patients results in (i) partial recovery of nucleus accumbens and other brain function, (ii) improvements in symptoms of depression and anxiety, and (iii) that persisting brain functional abnormalities in protracted abstinence patients correlate with clinical measures of negative affect and severity of drug use history.

## Materials and methods

### Participants

The study was approved by the East of Scotland Research Ethics Committee, reference number 06/S1401/32, and written informed consent was obtained from all participants. Thirty-three OUD male patients receiving MT were recruited from NHS Tayside Addiction Services. MT patients received regular urine tests for illicit drug use and all recruited patients had testing-confirmed abstinence of illicit drug use for six months prior to recruitment. Fifteen abstinent OUD patients (ABS group) were recruited from the Lothian and Edinburgh Abstinence Program (LEAP) and Phoenix Futures Scottish Residential Service Glasgow. These patients also received regular testing for covert drug use and had been abstinent for at least 6 weeks (range 6 weeks to 7 months). None of the methadone-maintained patients had previously lived in a residential abstinence unit. As patients were either stably maintained on methadone or were stably abstinent, no acute intoxication or withdrawal symptoms were observed. Both MT and ABS groups had been taking between 30 and 120 mg of methadone daily and had initially presented with more than 3 years of continuous daily illicit heroin use. The two groups were matched by lifetime drug use and methadone doses: initial titration of methadone dose (ITMD), current methadone treatment dose (CMTD), and/or last stable methadone dose (LSMD). The ITMD was the methadone dose required to abolish heroin withdrawal symptoms when initiating MT, which was between 10 and 30 mg daily identified using objective evidence of opioid withdrawal symptoms [16], reflecting the magnitude of physical dependence at the onset of MT. Twenty-three healthy age-matched males were recruited as controls.

Diagnoses were confirmed using the Mini International Neuropsychiatric Interview (MINI Plus, version 5.0) [17]. The MT group had an ICD10 304.01 diagnosis of Opioid type dependence, continuous, which corresponds to the current diagnosis of OUD in DSM 5. Mood and anxiety symptoms were assessed using the Hospital Anxiety and Depression Symptom (HADS) rating scale [18], cigarette use with the Fagerstrom scale [19], and IQ using the National Adult Reading Test (NART) [20]. Exclusion criteria were past or current histories of psychotic disorder, post-traumatic stress disorder, neurological and neurodevelopmental disorders, head injury, history of non-fatal overdose episodes, benzodiazepine, stimulant or alcohol dependence, and personality disorder (summary of screening and diagnostic clinical instruments in Supplemental Table 1). Ongoing abstinence from illicit drug use was confirmed prior to scanning using a multidrug urine test [21]. Details of participants are summarized in Table 1 and our previous publications [14, 22–25] (summary of cohort recruitment, treatment, and testing is provided in Supplemental Table 2). ABS patient data has not been reported previously.

**Table 1:** Demographic and clinical characteristics.

	MT	ABS	HC	Significance*
N	33	15	23	
Age^1^	33.9 (4.2)	37 (3.7)	30.8 (7.0)	ns
HADS-A	6.0 (4.4)	6.2 3.56	3.73 (3.8)	MT > HC *p* = 0.04 ABS > HC *p* = 0.05
HADS-D	4.2 (3.4)	3.0 (2.3)	1.65 (2.5)	MT > HC *p* = 0.002 ABS > HC ns (*p* = 0.1)
ITMD^2^	50 (19.0)	49.2 (40.3)		
SMD^2^	74.7 (18.8)	79 (36.0)	–	ns
Age1^st^ injecting opioids^1^	16.1 (3.5)	14.1 (3.6)	–	ns
Yrs of opioid use	9.1 (19.6)	3.8 (11.2)	–	ns
Age injecting opiods^1^	18.1 (8.0)	22.3 (7.0)	–	ns
Fagerström tot score	3.8 (1.9)	8.1 (13.0)	–	ns
Duration abstinence (days)	–	Between 6 and 7 months	–	–

Values are mean (SD). *ABS* abstinent group, *HADS-A* HADS Anxiety, *HADS-D* HADS Depression, *HC* healthy control group, *ITMD* initial methadone titration dose, *MT* methadone-treated group, *N* total number, *NART* National Adult Reading Test, *ns* not significant, *SMD* stable methadone dose, ^1^ = yrs=years; ^2^ = (mg/day); * *p*-values calculated for abstinent vs. control groups only, *mg* milligrams.

### Paradigm

**Fig. 2:**
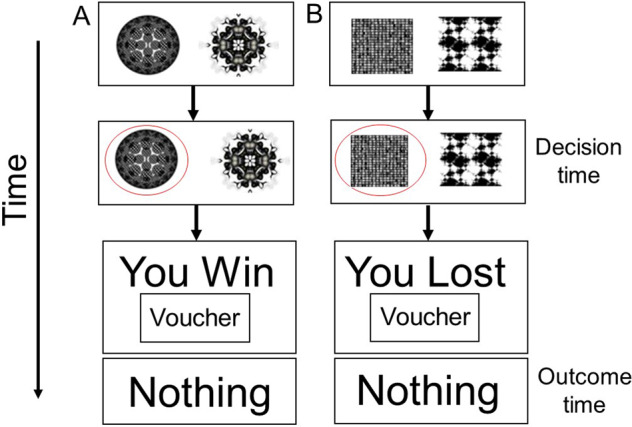
Behavioral paradigm. **A** Reward-gain (positive valence system) and **B** loss-avoidance (negative valence system) instrumental learning task.

Figure 2 shows the reward-gain and loss-avoidance instrumental learning task used during fMRI. We have previously used this task in fMRI studies of MT OUD patients [14], in binge drinkers with depression symptoms [5], and in patients with the treatment-resistant major depressive disorder [26]. The RDoC matrix includes ‘loss’ as an NVS construct and reward learning as a PVS construct [27]. Therefore, as with our prior study on binge drinking [5], brain responses to loss were considered measures of NVS and responses to reward the PVS.

Before scanning, all participants had a brief training session on the task on a PC, which used different stimuli than those used in the scanner. The task has three possible outcomes: rewarding (“win’), aversive (“lose”), and neither win nor lose (“nothing”) neutral. Volunteers were told that the aim of the task was to maximize winning and to avoid losing points (“vouchers”) as much as possible, and they had to learn to do this by trial and error. “Win trials” had two possible outcomes: “You Win” or “Nothing”. “Lose trials” had two possible outcomes: “You Lost” or “Nothing”. One pair of fractal images were associated with each type of outcome, and the association between a given pair of fractal images and win or lose outcomes were randomized across participants. The probability of win/loss fractal pairs had a fixed high probability (70%) and a fixed low probability (30%). Each session had 90 trials with each session lasting 13 min in total and 4 sessions per subject. The reward-gain and loss-avoidance trials were presented in a pseudo-random order.

### Image acquisition, pre-processing, and analyses

For each participant, functional whole-brain images were acquired using a 3 T Siemens Tim Trio scanner. A total of 37 slices were obtained per volume, with an echo planar imaging sequence comprising a repetition time (TR) of 2.5 s, echo time (TE) of 30 ms, flip angle 90°, the field of view 22.4 cm, matrix 64 × 64, with a voxel size of 3.5 × 3.5 × 3.5 mm.

Images were visually inspected for artifacts and pre-processed using Statistical Parametric Mapping (SPM) (http://www.fil.ion.ucl.ac.uk/spm/). First, images were realigned and co-registered to the SPM Montreal Neurological Institute echo-planar template. The average realigned co-registered image for each subject was then used to spatially normalize each realigned co-registered volume and smoothed with an 8 mm full width half maximum kernel. For a random-effects analysis, data from each subject were analyzed separately (first-level analyses) before summary statistical “beta” images were tested at the group level (second-level analyses). For testing NVS and PVS hypotheses, a first-level analysis was done comparing event-related activity at the outcome time for “loss” vs. “nothing” and “win” vs. “nothing” binary feedback events. For second-level random-effects analyses, summary statistical images from the first-level analyses for each subject were separately entered into second-level analyses to test for within-group activations/deactivations (one-group *t*-test) and between-group differences (ABS vs. controls; two group *t*-test). Significance was defined as *p* < 0.05 whole-brain, Family-wise error-corrected level, comprising a simultaneous requirement for a voxel threshold (*p* < 0.05) and a minimum cluster extent (120 voxels) identified using a commonly used Monte–Carlo method [28]. Region of interest analyses used SPM to extract the principal eigenvariate as the summary measure of brain response in 10 mm diameter spheres.

## Results

### Participants

HADS-A and HADS-D scores for the ABS group were 6.21 ± 3.56 and 3.00 ± 2.25 and for the control group 3.73 ± 3.83 and 1.65 ± 2.52, respectively. The ABS group was more anxious but not more depressed compared to healthy controls (Table 1). In the MT group the HADS-A and HADS-D scores, which were 6.00 ± 4.40 and 4.18 ± 3.38, respectively, were significantly higher than in the control group. No subjects met ICD10 criteria for mood or anxiety disorders.

### Behavioral analyses

Using a two-group t-test there were no significant differences between ABS and control groups for a total number of win events (*p* = 0.23) or a total number of loss events (*p* = 0.68).

### Negative valence system

**Fig. 3:**
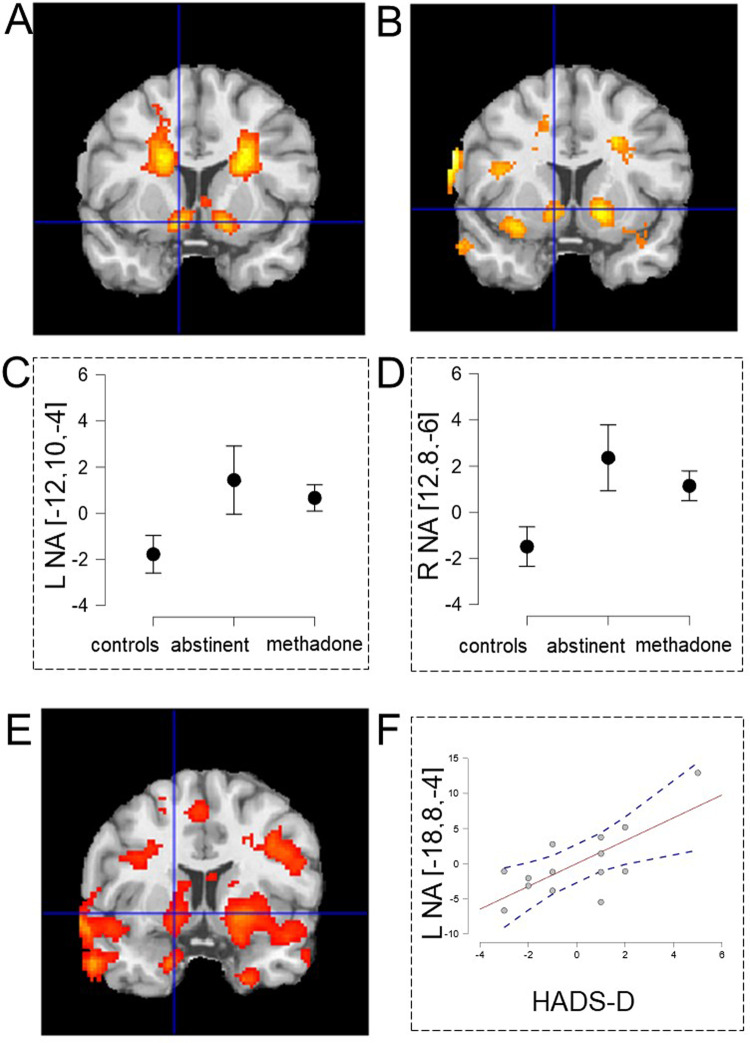
Negative valence system: brain responses to the feedback of unsuccessful loss-avoidance. **A** Deactivation of the nucleus accumbens in the control group and **B** significantly less bilateral nucleus accumbens deactivation in the abstinent group compared to controls. Previously we also reported significantly less accumbens deactivation in opioid-dependent patients receiving MT [14]. Region of interest centered at the maximally significant accumbens voxels in (**B**) illustrates accumbens deactivation in the three groups (**C**, **D**). Accumbens deactivation correlated significantly with increased HADS-D depression scores in the abstinent group (**E**) also illustrated using a region of interest (**F**) centered at the maximally significant accumbens voxel in (**E**). All brain regions significant at *p* < 0.05 whole-brain corrected.

**Fig. 4:**
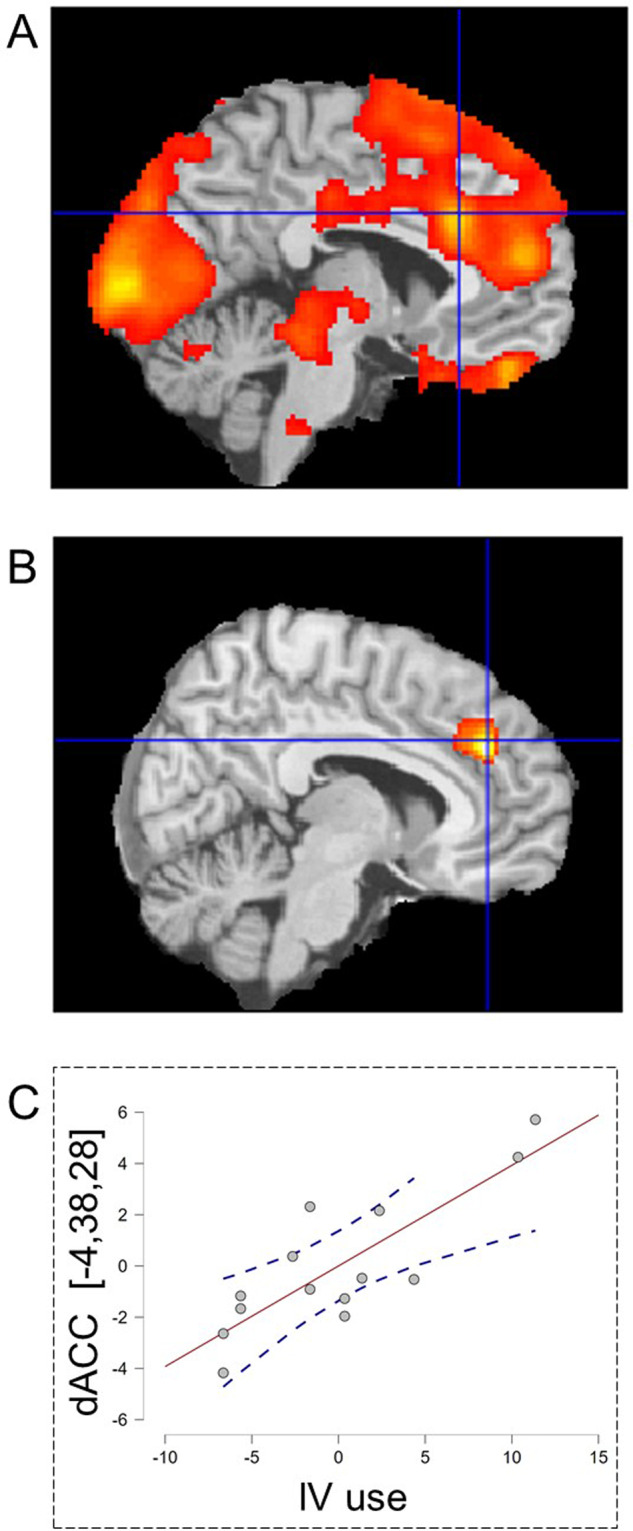
Negative valence system: brain responses to feedback to unsuccessful loss avoidance in abstinent patients. **A** The anterior mid-cingulate cortex activated in abstinent patients during unsuccessful loss avoidance, **B** this activation in patients positively correlated with years of intravenous (IV) drug use, also illustrated **C** with a region of interest centered on the maximally significant voxel in (**B**). All brain regions significant at *p* < 0.05 whole-brain corrected.

During loss events controls *deactivated* the nucleus accumbens (−12,10,−10) t = 3.81 and (12,8,−12) *t* = 3.71 and the caudate (−20,4,26) *t* = 4.57 and (22,4,24) *t* = 5.23 (Fig. 3A). ABS patients exhibited significantly less deactivation than controls in nucleus accumbens (12,8,−6) *t* = 3.00 and (−12,10,−4) *t* = 2.50 (Fig. 3B) and left insula (−32,14,−14) *t* = 3.58 (Fig. S1A, see Supplemental Table 3). Previously we reported significant failure of accumbens deactivation in MT OUD patients compared to controls [14] similar to ABS patients (Fig. 3C, D). Accumbens activation in ABS patients correlated positively with HADS-D ratings (Fig. 3E, F), such patients with more symptoms of depression had more activations, so were less like controls who strongly deactivated the accumbens and had minimal depressive symptoms. During loss events the ABS but not the control group strongly activated the anterior midcingulate cortex (aMCC)/dorsomedial prefrontal cortex (dmPFC) (−4,30,30) *t* = 7.42 (Fig. 4A). In ABS patients, years of intravenous (IV) drug use correlated with aMCC loss event activation (Fig. 4B, C). In summary, ABS patients showed blunted accumbens deactivation to loss event compared to controls similar to MT patients and an aMCC correlation with IV drug use.

### Positive valence system

During win events controls strongly activated the accumbens (−16,10,−10) t = 7.02; (16,10,−12) t = 6.94 (see Supplemental Table 4 and Supplemental Fig. 2A) and other regions including the amygdala-hippocampal complex (−30,−10,−22) *t* = 6.90; (20,−10,−22) *t* = 4.53) and posterior cingulate (4,−46,38) *t* = 4.73. Win event accumbens activations in ABS patients did not differ from controls (Fig. S2B) whereas it was significantly reduced in the MT group (−13,12,−12) *t* = 3.11 [14]. In ABS patients, accumbens win event activations correlated negatively with total days of methadone exposure, such that longer pre-abstinence exposure to methadone was associated with more blunting of accumbens activation (Fig. S2C, D). In summary, ABS patients showed accumbens win event activation similar to controls and unlike MT patients in whom reward activation was blunted.

## Discussion

Long-term abstinence is the desired goal for many OUD patients who have been stabilized on MT but one that is rarely achieved due to a very high relapse rate [1]. In the UK only 6–9% of OUD patients who had used heroin were abstinent at 33 months follow up with many of them having accessed residential rehabilitation programs [29], which is where we recruited the ABS patients for this study. Moreover, relapse following abstinence is associated with high overdose risks due to the loss of opioid tolerance [30]. This risk has been further exacerbated in the United States and Canada where potent synthetic opioids such as fentanyl have taken over the illicit drug market and been linked with high mortality rates [31]. As such, opioid detoxification as a strategy for the treatment of OUD is currently not recommended [2]. On the other hand, identifying characteristics associated with treatment success among abstinent previously opioid-dependent individuals could help guide personalized interventions for the treatment of OUDs.

Studies on protracted abstinence in animals with a history of dependence have emphasized blunted reward responses and increases in anxiety-like behavior persisting long after acute withdrawal, similar to but not as severe as during the dependent state [10]. Previously, we reported blunted PVS reward-linked accumbens activation and NVS loss-linked failure of accumbens deactivation in OUD patients receiving MT [14]. Here we recruited OUD patients previously treated with methadone who were stably abstinent for at least 6 weeks to test whether there was normalization of brain function and reductions in symptoms of depression and anxiety. Consistent with our hypotheses, we found accumbens reward-linked activation (PVS) was not blunted in ABS patients compared to controls, unlike MT patients [14]; ABS patients also reported fewer depressive symptoms than MT patients. However, ABS patients’ accumbens loss-linked deactivations (NVS) were blunted compared to controls, similar to MT patients [14]. Additionally, ABS patients, like MT patients, had significantly higher anxiety symptoms than controls. Our data showing partial recovery of nucleus accumbens function and reduction in depressive symptoms with protracted abstinence is consistent with predictions from the allostasis theory [6, 10, 32].

Here, we used fMRI to assess striatal function during stable abstinence in OUD. Previously with positron emission tomography (PET), we had consistently reported reduced striatal dopamine D2/D3 receptor availability in a variety of drug users compared to controls including individuals with heroin use disorder, alcohol dependency and cocaine and methamphetamine use disorders [33, 34]. A dopamine transporter (DAT) ligand PET study was used to measure pre-synaptic dopamine terminal function and reported reduced binding in MT OUD patients, which correlated with symptoms of anxiety and reduced striatal DAT binding, in protracted abstinence (6 months) OUD patients [35]. Similar reductions in DAT binding have been reported in a variety of other drug addictions [36]. Our instrumental fMRI task is a modified version of the Pessiglione task in which brain activity reflects, in part, dopamine function [37]. Blunted reward gain (PVS) accumbens activation in MT patients (but not ABS patients) measured using fMRI, which we use as an indirect measure of striatal dopamine function [14], might therefore be accounted for by blunted dopamine release [35]. This suggests MT maintains a state of decreased reward sensitivity in OUD patients whereas protracted abstinence might facilitate recovery of reward sensitivity and reduce depression. However, the directionality of the association cannot be discerned in our study for it is also possible that OUD patients who had less PVS disruption were those who were able to maintain abstinence. Thus, longitudinal studies of protracted abstinent patients are needed to establish whether reward sensitivity recovers, or to determine whether recovery of PVS function might serve as a potential biomarker of when OUD patients may be able to discontinue medication for OUD. For such studies and in general, a strategy to reduce the risk of overdoses in OUD patients who seek detoxification from methadone or buprenorphine is to consider treatment with the opioid receptor antagonist naltrexone (extended-release Naltrexone or Vivitrol) [38].

Two parallel routes through the basal ganglia, the direct and indirect pathways, have long been recognized [39]. Preclinical studies have shown that positive reinforcement is linked to accumbens dopamine D1 receptor activation of the direct pathway; negative reinforcement to deactivation of accumbens D2 receptors in the indirect pathway [40]. Here we found abnormally blunted accumbens deactivation during negative reinforcement (loss avoidance) in ABS patients similar to our previous findings in MT OUD patients [14], which could reflect reduced striatal D2 receptor availability [33, 34] and be associated with increased vulnerability to relapse [41]. However, studies that concomitantly measure fMRI and PET are required to test this hypothesis and the link between abnormal accumbens deactivation and depression symptoms is unclear. Notably, though, we found anterior insula activation with loss events in abstinent patients and there is preclinical evidence that the insula may drive hyperkatifeia via the amygdala in alcohol dependence [42].

Using the same fMRI task in binge drinkers, we reported aMCC/dmPFC activation during loss events, which correlated with years of alcohol use [5]. In the present study, we also report loss event aMCC/dmPFC activation that correlated with years of pre-abstinence IV heroin use, and striatal deactivation in ABS OUD patients. Interestingly, the aMCC/dmPFC is strongly implicated in negative affect, cognitive control, and experience of pain [43, 44] (part of the NVS) and heightened evoked pain responses might be compensatory responses during an aversive state [45]. Therapeutic lesions in the aMCC (anterior cingulotomy) have been used to treat OUD [46] and alcohol dependence [47]. Connectivity studies of this region are indicated.

Hyperkatifeia, which is defined as a negative emotional state that includes increased anxiety and stress vulnerability, depressive symptoms, the elevation of reward thresholds, and lower pain thresholds is associated with OUD [9]. This aversive experience is represented by an enhancement of the “b process” and a downward shift in baseline mood (Fig. 1B) [9]. In our study, we also found that the anterior insula, which is involved with interoception [48] activated with loss events in ABS patients. This suggests that enhanced awareness of the aversive effects of loss might contribute to hyperkatifeia. Indeed insular activation correlated positively with the initial titration dose of methadone (Fig. S1), which we had previously reported predicted whether an MT OUD patient achieved abstinence [22]. Further, the somatic marker theory of addiction proposed that the insula is involved in drug withdrawal/deprivation [49] and insula stroke eliminated cigarette craving [49]. The anterior insula is strongly interconnected with the aMCC/dmPFC with these locations tending to co-activate with aversive events [50]. Thus, the results from our present study suggest that OUD may have long-term effects on insula stress responses that persist even after protracted abstinence.

Limitations of this study include a small sample size, which reflects the difficulties in recruiting OUD patients who have been able to successfully discontinue MT, due to the high relapse rate associated with MT discontinuation. Other limitations are that we only recruited males and thus these findings may not generalize to ABS and MT female populations. We used a cross-sectional design so we cannot disentangle pre-drug use vulnerability factors from the effects of OUD, although our results are consistent with predictions from pre-clinical animal studies, which do not have this limitation. Notably, we cannot rule out the possibility that individuals who are less able to deactivate nucleus accumbens with negative reinforcers (e.g., loss of money) are more vulnerable to drug use and subsequent OUD. Nor can we rule out the possibility that recovery of PVS accumbens activation might have allowed OUD patients to successfully discontinue methadone treatment. Also, our ABS OUD patients have been abstinent for a period that ranged between six weeks to seven months so it is unclear if longer abstinence might have led to the recovery of NVS processes. Future larger studies on protracted abstinence patients using a longitudinal design are indicated.

In summary, consistent with allostasis theory predictions from preclinical and human brain imaging studies, we found evidence for abnormal striatal brain responses in protracted abstinence OUD patients. Our findings show preliminary evidence of recovery of the PVS in OUD patients whereas this was not the case in OUD patients on MT. Abstinence in individuals with OUD presents serious risks of fatal overdose on relapse, due to the rapid loss of tolerance that occurs during even brief periods of abstinence. This risk is particularly acute in countries where fentanyl is readily available: e.g., drug deaths have recently exceeded 100,000/year in the USA being further exacerbated by the COVID-19 pandemic [51, 52]. Our findings also highlight the importance of the NVS as a target for new OUD treatments (e.g., partial MOR1 agonism, k-type opioid receptor antagonism, and α-2A adrenergic receptor agonism) [53, 54].

## Supplementary information

Supplemental Material

## Data Availability

The data that support the findings of this study are available from the corresponding author, upon reasonable request.
