# Supplementary material for: Protracted abstinence in males with an opioid use disorder: partial recovery of nucleus accumbens function
Source: Transl Psychiatry. 2022 Feb 26;12:81. doi: 10.1038/s41398-022-01813-4 (PMC8881207; doi:10.1038/s41398-022-01813-4)
Supplement: Supplementary file 1 — Supplemental Material [file 41398_2022_1813_MOESM1_ESM.docx]

**SUPPLEMENTAL MATERIAL**

**Supplemental Table 1.** Summary of screening and diagnostic of the clinical instruments and their measures.

**Supplemental Table 2.** Summary of cohort recruitment, treatment, and testing.

**Supplemental Table 3.** Within group activations and between group comparisons for Negative Valence System (‘Loss events’).

**Supplemental Table 4.** Within group activations and between group comparisons for Positive Valence System (‘Win events’).

**Supplemental Figure 1.** Negative Valence System: Insula.

**Supplemental Figure 2.** Positive valence system.

**Supplemental Table 1.** Summary of screening and diagnostic clinical instruments and their measures/domains.

| **Screening Tests** | **Measures/Domains** |
| --- | --- |
| Mini International Neuropsychiatric Instrument (MINI) *v5.0.* | Diagnosis of 15 Axis 1 and 1 Axis 2 DSM-4 psychiatric illnesses and substance misuse/dependence presented in 24 modules. |
| Case records from Addiction, Psychiatric and General Practitioner’s Services. | Identification of non-fatal overdose episodes  Confirming a history or not of epilepsy and other neurological phenomenon including learning disabilities.  Confirming a diagnosis of Hepatitis B, C and HIV  Validating medical and psychiatric histories  Validating substance misuse career and current drug and alcohol use.  Scottish Index of Multiple Deprivation (SIMD).  Calculating morphine equivalent dosing  Validating medication history |
| Post Trauma Amnesia Questionnaire *v1.0.* | Information on head and other cerebral insults and consequential post trauma amnesia. |
| Urine analysis for drug screen. | Identify presence of amphetamine, cannabinoids, opioids, benzodiazepine and cocaine. |
| **Diagnostic Tests** | **Measures/Domains** |
| Mini International Neuropsychiatric Instrument (MINI) (Section L) *v5.0.* | Diagnosis of opioid misuse/dependence (lifetime and current). |
| Maudsley Addiction Profile (MAP) *v1.0.* | Information on substance misuse history, health risk behaviour, physical and psychological health, personal and social functioning in the last 30 days. |
| Fagerström Test for Nicotine Dependence (FTND) *v1.0.* | 6 items with a diagnostic score ranging from 0 (no nicotine dependence) to 10 (very severe nicotine dependence). |
| Clinical Opiate Withdrawal Scale (COWS) *v1.0.* | 11 objective and subjective symptoms of opioid withdrawal. Scores ranged from 0-48. |
| Urine analysis for drug screen. | Urine drug test with the sample tested through an automated enzyme-mediated immunoassay |

**Supplemental Table 2.** Summary of cohort recruitment, treatment, and testing

| **Patient selection and enrolment** | **MT** | **ABS** | **HC** |
| --- | --- | --- | --- |
| **Identifying Recruits** | Male opioid dependent individuals aged over 18 years, on a structured methadone  treatment programme for more than 6 months. The mean daily dose of  methadone taken by this group varied but was within the 40-120 mg range | Male individuals aged over 18 years, who had previously engaged with a structured methadone. Individuals had been abstinent from all opioids for more than 2 months before being approached for recruitment. | Males aged over 18 years, who had no history of illicit drug use or a lifetime continuous/regular prescription of opiates.  Individuals were recruited opportunistically from the general population where the other study cohorts’ participants were residing. |
| **History of illicit drug use prior to recruitment – period of illicit drug use prior to recruitment, history of relapse during treatment, period of stabilisation prior to recruitment** | The participants had self-reported histories of more than three years of  regular, daily illicit opioid (usual heroin) use and met a diagnosis of opioid  dependence syndrome according to DSM-IV.  On average each individual had historically experienced 2-3 episodes of ceasing illicit drug use before relapsing back into daily illicit drug use.  Participants had been maintained on methadone for 15 months +/- 6 months prior to recruitment and had ceased illicit drug use for at least 6 months. | The participants had been previously engaged in an opioid treatment programme. This was confirmed by reviewing their medical records. Prior to engaging with ORT, individuals self-reported histories of more than three years of regular, daily illicit opioid (usually heroin) use and met a diagnosis of opioid  dependence syndrome according to DSM-IV.  On average each individual had historically experienced 2-3 episodes of ceasing illicit drug use before relapsing back into daily illicit drug use.  All participants had remained avoidant of opioids for at least 6 weeks prior to recruitment. | No history of illicit drug use or a lifetime continuous/regular prescription of opiates |
| **Confirmation of drug history prior to recruitment** | Stabilisation on ORT for 6 months prior to recruitment was confirmed by a retrospective review of clinically obtained UDS, a patient’s self-reported history and the assessment of the individual’s treating clinician. | Previous engagement with an ORT programme was confirmed by reviewing their medical records.  History of at least a 2-month period of discharge from a treatment programme was also confirmed by reviewing their medical records. |  |
| **Assessment of psychological history prior to recruitment** | Individuals were excluded from the study if they had past or current diagnoses of psychotic disorder, post-traumatic stress disorder, antisocial and borderline personality disorders. Individuals were screened for current/past psychological history using the Mini International Neuropsychiatric Interview and by reviewing medical notes  Individuals were excluded from the study if they reported experiencing an overdose that led to an admission to hospital |  |  |
| **Assessment of overdose history prior to recruitment** | Participants were asked to self-report their overdose history at the recruitment visit.  Individuals were excluded from the study if they reported experiencing an overdose that led to an admission to hospital. |  |  |
| **Screening undertaken to confirm eligibility at study visits** | At study visits individual’s self-reported no on-going illicit drug use and provided UDT to confirm this. | At study visits individual’s self-reported no on-going illicit drug use and provided UDT to confirm this. | At study visits individual’s self-reported no on-going illicit drug use and provided UDT to confirm this. |
| **Timing of ORT consumption prior to study visits** | Participants reported taking their daily dose of methadone prior to all study visits. | Not applicable to this study cohort. | Not applicable to this study cohort. |

H= Heroin group, MT= Methadone Treatment group, ABS=Abstinent group, HC= Healthy Control group; UDT; ORT=Opioid Replacement Treatment; UDT=Urine Drug Testing.

**Supplemental Table 3.** Within group activations and between group comparisons for Negative Valence System (‘Loss events’).

|  | **x** | **y** | **z** | **T** |
| --- | --- | --- | --- | --- |
| **Controls** |  |  |  |  |
| L posterior midbrain | -8 | -22 | -6 | 3.83 |
| Dorsal anterior cingulate cortex | 2 | 28 | 24 | 4.57 |
| Frontal lobe, medial frontal gyrus | 2 | 52 | 18 | 3.95 |
| L frontal lobe, inferior frontal gyrus | -34 | 30 | -22 | 4.73 |
| L temporal lobe, superior temporal gyrus | -42 | 20 | -32 | 4.35 |
| R frontal lobe, inferior frontal gyrus | 40 | 24 | -22 | 5.35 |
| R temporal lobe, superior temporal gyrus | 34 | 22 | -36 | 5.71 |
| Frontal lobe, superior frontal gyrus | -8 | 16 | 68 | 5.04 |
| L frontal lobe, precentral gyrus | -50 | -2 | 52 | 4.03 |
| L occipital lobe | -12 | -96 | 12 | 7.97 |
| R occipital lobe | 12 | -100 | 6 | 7.85 |
| Cerebellum | 36 | -40 | -28 | 3.67 |
| Cerebellum | -22 | -64 | -12 | 3.59 |
|  |  |  |  |  |
| **Abstinent** |  |  |  |  |
| Dorsal anterior cingulate cortex | -4 | 30 | 30 | 7.42 |
| Posterior cingulate cortex | 0 | -18 | 40 | 6.08 |
| L insula | -36 | 16 | -12 | 6.44 |
| R insula | 40 | 16 | -6 | 5.39 |
| L midbrain | -6 | -30 | -2 | 5.69 |
| R midbrain | 4 | -32 | -2 | 5.72 |
| L occipital lobe | -12 | -96 | 12 | 6.18 |
| R occipital lobe | 12 | -100 | 6 | 5.92 |
|  |  |  |  |  |
| **Controls > Abstinent** |  |  |  |  |
| L medial frontal gyrus | -26 | 42 | 12 | 3.25 |
| R medial frontal gyrus | 18 | 44 | -12 | 3.12 |
|  |  |  |  |  |
| **Abstinent >Controls** |  |  |  |  |
| R nucleus accumbens | 12 | 8 | -6 | 2.93 |
| L nucleus accumbens | -12 | 10 | -4 | 2.45 |
| L brainstem | -12 | -28 | -28 | 3.33 |
| L posterior cingulate | -16 | -38 | 10 | 3.70 |
| R anterior hippocampus gyrus | 20 | -42 | 10 | 3.06 |
| L insula | -32 | 14 | -14 | 3.58 |

Coordinates (x, y, z) reported in MNI space; R/L*=*right/left. All results significant at p<0.05 cluster extent corrected across the whole-brain.

**Supplemental Table 4.** Within group activations and between group comparisons for Positive Valence System (‘Win events’).

|  | **x** | **y** | **z** | **T** |
| --- | --- | --- | --- | --- |
| **Controls** |  |  |  |  |
| L nucleus accumbens | -16 | 10 | -10 | 7.02 |
| R nucleus accumbens | 16 | 10 | -12 | 6.94 |
| L dorsal caudate | -18 | 20 | 12 | 5.10 |
| Midbrain | 0 | -20 | -12 | 3.69 |
| Thalamus | 6 | -6 | 0 | 4.52 |
| Medial prefrontal cortex | 0 | 46 | 2 | 6.64 |
| L amygdala-hippocampal complex/PHG | -30 | -10 | -22 | 6.90 |
| R amygdala-hippocampal complex/PHG | 20 | -10 | -22 | 6.93 |
| L insula | -40 | 0 | -4 | 3.40 |
| R insula | 38 | 2 | -2 | 4.17 |
| Posterior cingulate cortex | 4 | -28 | 32 | 6.45 |
| L occipital lobe, cuneus | -16 | -102 | 16 | 13.69 |
| R occipital lobe, middle occipital lobe | 26 | -94 | 10 | 14.39 |
| L cerebellum | -40 | -62 | -40 | 5.47 |
| R cerebellum | 42 | -60 | -40 | 5.31 |
|  |  |  |  |  |
| **Abstinent** |  |  |  |  |
| L nucleus accumbens | -14 | 10 | -8 | 5.92 |
| R nucleus accumbens | 10 | 8 | -12 | 5.98 |
| R Subgenual cingulate cortex | 2 | 34 | 4 | 4.99 |
| L dorsal anterior cingulate cortex | -8 | 38 | 20 | 4.17 |
| R dorsal anterior cingulate cortex | 16 | 36 | 16 | 3.58 |
|  |  |  |  |  |
| **Controls > Abstinent** |  |  |  |  |
| R thalamus | 2 | -2 | 8 | 2.94 |
| L amygdala | -34 | -8 | -20 | 3.49 |
| R amygdala | 24 | -14 | -18 | 2.94 |
|  |  |  |  |  |
| **Abstinent >Controls** |  |  |  |  |
| Medial Prefrontal cortex | 0 | 32 | 1 | 3.17 |

Coordinates (x, y, z) reported in MNI space; R/L*=*right/left. All results significant at p<0.05 cluster extent corrected across the whole-brain.


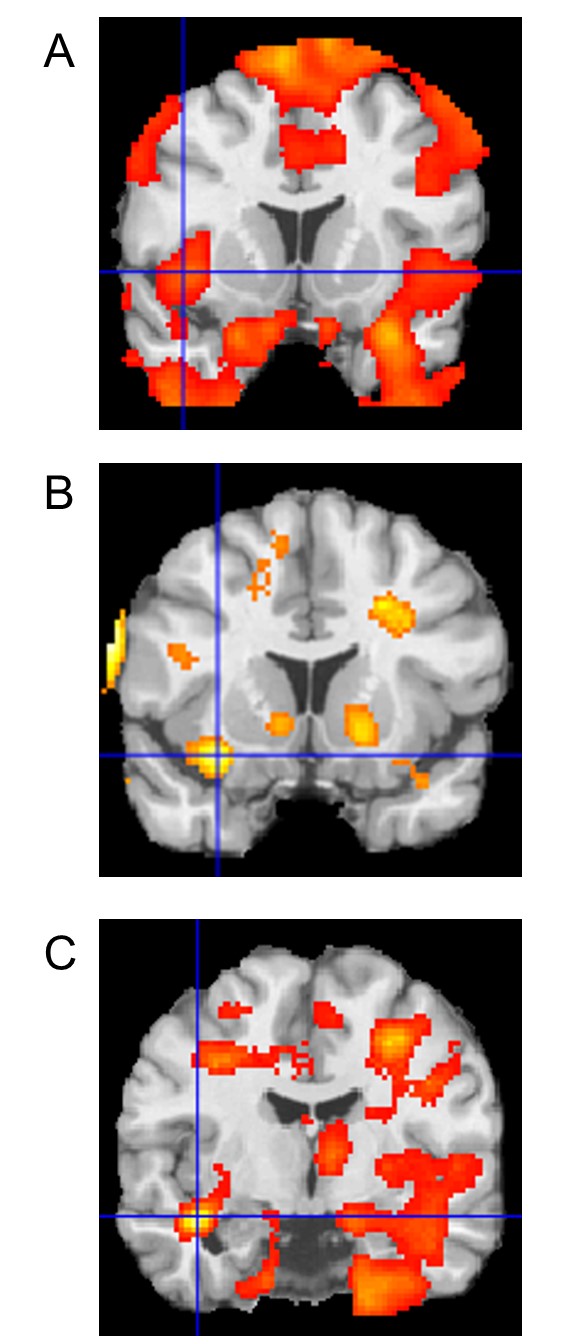


**Supplemental Figure 1.** Negative Valence System

Brain responses to feedback to unsuccessful loss avoidance in abstinent: (A) the insula activated in abstinent during unsuccessful loss avoidance; (B) significantly less insula deactivation in the abstinent group compared to controls, which positively correlated with the first dose of methadone (C).


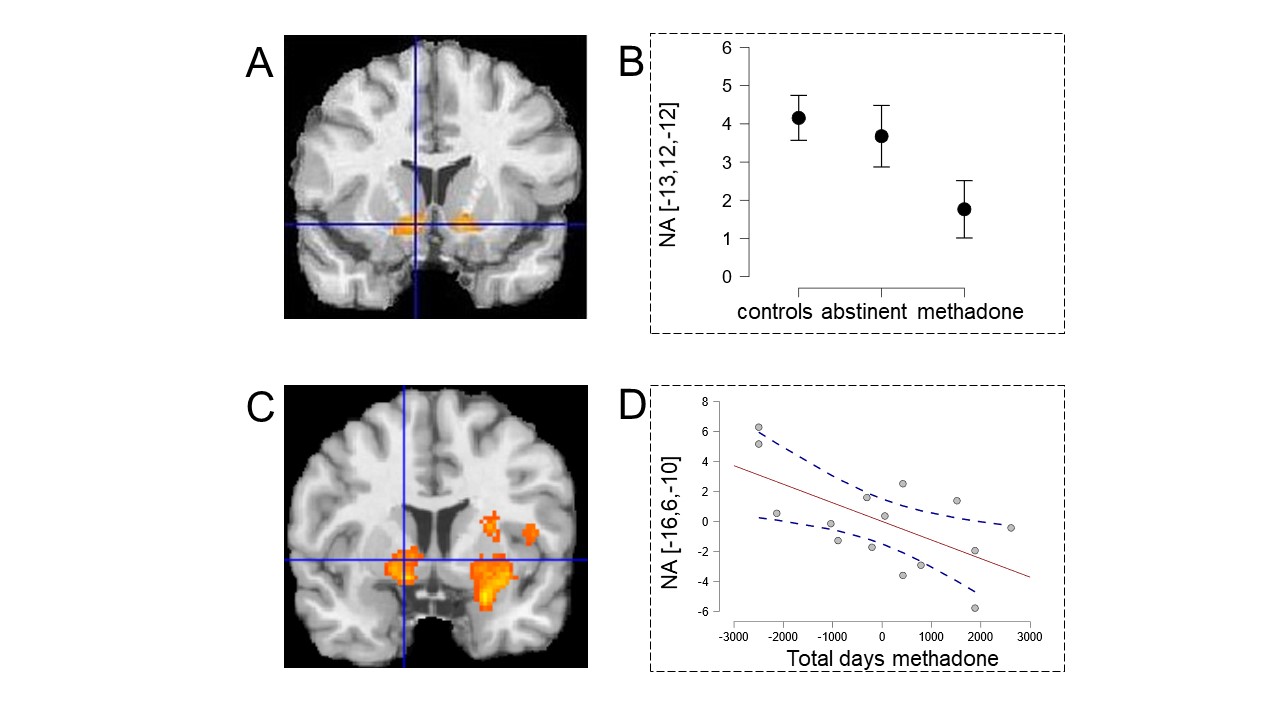


**Supplemental Figure 2.** Positive valence system. (A) Accumbens activation to win events in healthy controls. We have previously reported blunted accumbens reward activation in opioid dependent patients receiving MT (Gradin et al, 2014). Abstinent patients did not have significantly reduced win event activation compared to controls. (B) Accumbens activation in the three groups illustrated using a region of interest centered at the maximally significant voxel in (A). In abstinent patients accumbens win event activation correlated negatively with lifetime days of methadone (D) which is also illustrated (D) using a region of interest centered at the maximally significant voxel in (C). All brain regions significant at p<0.05 whole brain corrected.
